# A Triadic Reflective-Impulsive-Interoceptive Awareness Model of General and Impulsive Information System Use: Behavioral Tests of Neuro-Cognitive Theory

**DOI:** 10.3389/fpsyg.2016.00601

**Published:** 2016-04-26

**Authors:** Ofir Turel, Antoine Bechara

**Affiliations:** ^1^Department of Information Systems and Decision Sciences, California State UniversityFullerton, CA, USA; ^2^Department of Psychology, Brain and Creativity Institute, University of Southern CaliforniaLos Angeles, CA, USA

**Keywords:** problematic information technology use, planned behavior, satisfaction, impulsive behavior, temptation, reflective-impulsive model, dual-systems, interoceptive awareness

## Abstract

This study examines a behavioral tripartite model developed in the field of addiction, and applies it here to understanding general and impulsive information technology use. It suggests that technology use is driven by two information-processing brain systems: reflective and impulsive, and that their effects on use are modulated by interoceptive awareness processes. The resultant reflective-impulsive-interoceptive awareness model is tested in two behavioral studies. Both studies employ SEM techniques to time-lagged self-report data from *n*_1_ = 300 and *n*_2_ = 369 social networking site users. Study 1 demonstrated that temptations augment the effect of habit on technology use, and reduce the effect of satisfaction on use. Study 2 showed that temptations strengthen the effect of habit on impulsive technology use, and weaken the effect of behavioral expectations on impulsive technology use. Hence, the results consistently support the notion that information technology users' behaviors are influenced by reflective and impulsive information processing systems; and that the equilibrium of these systems is determined, at least in part, by one's temptations. These results can serve as a basis for understanding the etiology of modern day addictions.

## Introduction

Neurocognitive models of problematic and addictive behaviors have traditionally relied on the dual-system perspective for explaining the etiology of such behaviors (Everitt et al., 2001). According to this perspective, problematic and addictive behaviors stem from (1) increased activity of an impulsive brain system, which mediates the generation of impulsions to act rashly and automatically in order to obtain implicitly-associated incentive rewards; and (2) diminished ability of a reflective brain system to inhibit the generated impulsions (Turel et al., 2014).

Recent models of the neurocognitive basis of problematic and addictive behaviors suggest that a third system, interoceptive awareness, may be at play (Naqvi et al., 2007). This system can lead people to occasionally sense temptations, i.e., conscious momentarily salient desires to engage in a rewarding behavior (e.g., use substances, smoke, or use an information system). Impairment of this system can eliminate such desires (Naqvi et al., 2007). The temptations which are relayed through this system violate users' homeostasis and require attention and possibly action. However, the selected action is often rash and relies on the impulsive system, because interoceptive awareness activations can weaken the inhibition and reflection abilities of the reflective system as they temporarily occupy and impair it; and can strengthen the influence of the impulsive system by exciting it (Noël et al., 2013).

The neuroanatomical and neurobiological elements of these systems have been largely identified in prior research. Much of the reflective information processing takes place at the prefrontal cortex which integrates information from various sources, uses conscious reflections, and makes behavioral decisions, which are then transferred to motor cortices for execution (Bechara et al., 1999). Thus, the reflective system is mainly prefrontal cortex dependent (Bechara et al., 2000b). In contrast, the impulsive system is largely amygdala-striatum (dopamine) dependent and it is essential for forming habituated and automatic behaviors (Yin et al., 2004; Yin and Knowlton, 2006). This system promotes such behaviors through learning and association-building (Wingard and Packard, 2008; Popescu et al., 2009). In extreme cases, hyper-sensitizing this system can cause habits to transition into addictions and problematic behaviors (Turel et al., 2014). Lastly, the interoceptive awareness system is insular cortex (“insula”) dependent. The insula serves as a gate to visceral needs and mediates the generation of homeostatic perturbations, like the sense of temptation, withdrawal, and stress (Craig, 2009). Insular activity can promote the execution of motivational states by determining the effectiveness of incentive inputs and diverting the attention of people from reflection; feedback loops signaling the status of the viscera adjust insular activity and consequent influences on impairment of reflection processes and sensitization of impulsive ones (Noël et al., 2013).

Behavioral and neurocognitive research regarding this tripartite model of human behavior is scarce. This study seeks to partially bridge this gap and behaviorally test a reflective-impulsive-interoceptive awareness model (RIIAM) of human behavior. We do so in the context of social networking sites (SNS) given their growing potential to facilitate problematic and addictive behaviors (Turel et al., 2014; Turel, 2015b; Turel and Bechara, 2016). Non-chemical addictions and especially the problematic use of hedonic information systems (IS) such as videogames and social networking sites, have gained increased recognition given their potential harms to individuals and societies (Turel et al., 2011a,b). For instance, the fifth edition of the Diagnostic and Statistical Manual for Mental Disorders includes “Internet Gaming Disorder” in Section 3 (emerging concepts that require additional research; American Psychiatric Association, 2013). Similarly, problematic use of SNS, the symptoms of which resemble those of other behavioral addictions (Andreassen et al., 2012), is on the rise. Key symptoms of these potential disorders include impulsive, less-planned, excessive, and possibly deviant (that deviates from the law or heightens the risk to encounter negative consequences) and problematic use patterns of the IS (Turel, 2015b). Understanding cognitive manifestations and associated brain etiology that sub-serve such behaviors is therefore an important endeavor, which can help with the development of a broader knowledge base regarding the cognitive and neurocognitive basis of problematic information technology use behaviors, as well as with the development of intervention programs.

Because it is practically impossible to account for all elements or associated brain activations of the three abovementioned brain systems, this manuscript describes two separate time-lagged studies and focuses on one key perception or state with which each system is presumed to be involved, in each one of the studies. In the first study, the outcome variable is SNS use, and in the second study the outcome variable is impulsive SNS use defined as spontaneous use without thinking or planning to do so (Rook and Fisher, 1995). In both studies it is first argued that IS users follow a reasoned and reflection-based use pattern, in which their satisfaction with system use (Study 1) and behavioral expectations regarding impulsive use (Study 2) predict their future IS use behaviors. Satisfaction is a positive cognitive-affective reaction to the IS; it is assumed to be associated with the reflective system since it is based on a mindful reflection whether the expectations from the system are met (Turel and Serenko, 2006). Similarly, behavioral expectation is a subjective assessment of the likelihood that a person will use the system spontaneously, without planning in the near future (next week in our case); the reflective brain system is likely to be implicated in processing such expectations since this is a higher-order task which requires forward-looking integration of data from multiple sources.

The impulsive system is operationalized in both studies with measures of SNS use habit (uni-dimensional in study 1 and multidimensional in study 2). The role of the impulsive system and especially the striatum in habit formation, maintenance and execution is well-established (Yin et al., 2004). Lastly, both studies suggest that the temptations users feel in various situations weaken their ability to rely on the reflective system (satisfaction; behavioral expectations); and drive them to depend more heavily on the impulsive system (habit). Moreover, temptations can also exert a direct effect on system use, which is likely mediated through reflective and impulsive processes not captured in this study. Temptations were chosen as plausible manifestation of the interoceptive awareness system because they represent common and frequent visceral disturbances that require attention and can “hijack” decision making processes (Baumeister, 2002).

**Figure 1:**
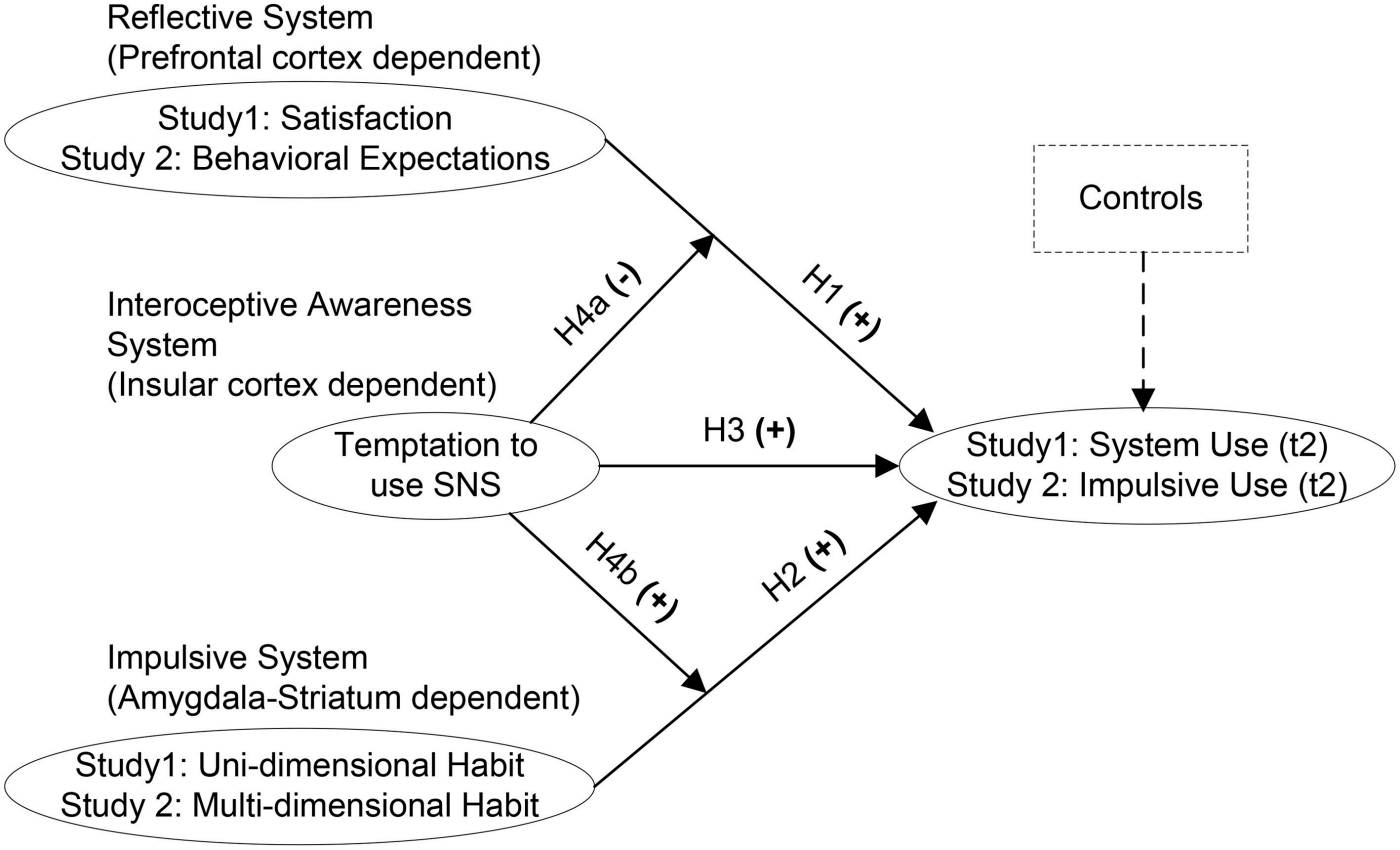
**Research model**.

Integrating these views, we propose the following hypotheses and model (see Figure 1).

**H1:** Satisfaction with system use (study 1) and behavioral expectations regarding impulsive system use (study 2) will be positively associated with system use (study 1) and impulsive system use (study 2).
**H2:** System use habit will be positively associated with system use (study 1) and impulsive system use (study 2).
**H3:** Temptation to use an IS will be positively associated with system use (study 1) and impulsive system use (study 2).
**H4:** Temptation is associated with disequilibrium between the reflective and impulsive information processing systems
  **(a)** Temptation weakens the effect of satisfaction (study 1) and behavioral expectation (study 2) on system use.
  **(b)** Temptation strengthens the effect of habit on system use.

## Study 1

### Methods

#### Participants

Three hundred SNS users who were university students provided complete responses (net response rate of 77% from all invitees, and 81% from SNS users). The sample was equally split between man (50%) and women and users' average age was 22.8 years (18–46, median = 22, *SD* = 3.72). They had an average of 428.7 contacts on their SNS (2–3553, median = 350, *SD* = 478) and, on average they had 4.54 years of experience with it (0.25–10 years, median = 4, *SD* = 2.77). The majority of the sample was Facebook users (87%), followed by LinkedIn users (8.7%) Google+ users (2.3%), and Twitter users (2%). Thus used, on average, 3.5 SNSs (1–8, median = 2, *SD* = 1.75).

#### Procedure and materials

Data were collected with two online surveys administered at two points in time, 1 week apart, to undergraduate students taking a business class at a large American university. Participation was voluntary in exchange for bonus points and the study's protocol was approved by the Institutional Review Board of California State University, Fullerton. Because many participants have used multiple SNSs, both surveys asked individuals to point to the one SNS they use most frequently and reflect and report on its use. The survey instruments included scales which were adapted from well-established and reliable measures (see scales, sources and reliabilities in Table 1). It also included age, gender (male = 1), number of contacts a user has on the SNS, GPA (seven-point scale, from 1 = “< 2.4” to 7 = “3.9–4.0”) and experience (years on the selected SNS) as controls. It was pretested and validated (see Appendix A in Supplementary Materials).

**Table 1:** **Study 1 measures and reliabilities**.

	**Sources**	**Items**	
Survey 1 (*t*_1_)	Satisfaction (Bhattacherjee, 2001) α = 0.86	How do you feel about your overall experience of using this social networking website?	
		-Dissatisfied (1) to Satisfied (7)	
		-Displeased (1) to Pleased (7)	
		-Frustrated (1) to Contented (7)	
		Terrible (1) to Delighted (7)	
	Habit (Turel and Serenko, 2012) α = 0.78	[1 = Strongly disagree, 7 = Strongly agree]	
		-Using this social networking website has become automatic to me	
		-Using this social networking website is natural to me	
		-When I want to interact with friends and relatives, using this social networking website is an obvious choice for me	
	Temptation (Second-order construct, based on Velicer et al., 1990; Gwaltney et al., 2001) α = 0.72	Listed below are situations that lead some people to use their social networking website. We would like to know HOW TEMPTED you may be to use this website in each situation. [1 = Not at all tempted, 5 = Extremely tempted]	
		Temptation in positive affect situations α = 0.79	-When meeting with my friends
			-With my family or close friends who are using the social networking website
			-When I am happy and celebrating
			-When I see someone using a social networking website and enjoying it.
			-Over coffee while talking and relaxing
			-With friends at a party
		Temptation in negative affect situations α = 0.83	-When things are not going the way I want and I am frustrated
			-When I am very angry about something or someone
			-When I am extremely anxious and stressed
			-When I wake up in the morning and face a tough day
		Temptation in craving situations α = 0.71	-When I desire to check my social networking website
			-When I realize that stopping to use the social networking website is an extremely difficult task for me
			-When I first get up in the morning
			-When I feel I need a lift
			-When I realize I haven't checked my social networking website for a while
		Temptation in boredom/ idle time situations α = 0.71	-When I am home alone and have nothing to do
			-When life seems to be too boring
			-When I am stuck in traffic
			-When at work and have a short break
Survey 2 (*t*_2_)	System use (based on items in Turel, 2015a) α = 0.76	Over the last week…(1 = very low, 7 = very high)	
		-What was the extent of your use of your social networking website in terms of time you spend on it?	
		-What was the extent of your use of your social networking website in terms of the frequency (how often) you use it?	
		-Overall, how do you consider the extent of your social network website use over the previous week?	

#### Data analysis

Data were preliminarily analyzed with SPSS 23, and then the proposed model was estimated with AMOS 23 (a Structural Equation Modeling package). Structural Equation Modeling (SEM) is a technique which allows the simultaneous estimation of the factor loadings and path coefficients describing a theory-based complex network of relationships. It was chosen in this study because our theory involves multiple latent constructs. SEM models are typically assessed in two steps (Anderson and Gerbing, 1988). First a confirmatory factor analysis (CFA) model in which all constructs are modeled and allowed to freely correlate is estimated. This allows the assessment of convergent and discriminant validities of the latent variables. Next, a structural model is estimated in which the paths between the constructs are modeled as per the proposed theory. The overall ability of an SEM model with its estimated path coefficients to reproduce a given data structure is assessed by means of various fit indices which assess the gap between the observed and implied correlation matrices. Typical cutoff criteria for judging the goodness-of-fit of a model are given in Hu and Bentler (1999). For more detail on these techniques, readers my may refer to Kline (2010).

### Results

Several preliminary analyses were performed (See details in Appendix B of Supplementary Materials). First, multivariate analysis of variance ruled out possible SNS-based differences. Second, a correlation table was generated, and measures of construct reliability and internal consistency (Cronbach's alpha, composite reliability, and Average Variance Extracted) were calculated for the latent constructs. They suggested sufficient reliability and convergent validity (Hair et al., 1998).

After the preliminary tests, a CFA model was estimated with AMOS 23. This model included satisfaction, habit, system use, and temptation. The model presented good fit [$\chi_{\left(70\right)}^{2}$ = 137.1, CFI = 0.96, IFI = 0.96, RMSEA = 0.057 with p-close = 0.21, and SRMR = 0.048]. All loadings were significant (*p* < 0.001). These indices further established reasonable convergent and discriminant validities of the measurement model; and implied that it is possible to proceed to the assessment of the structural model.

**Figure 2:**
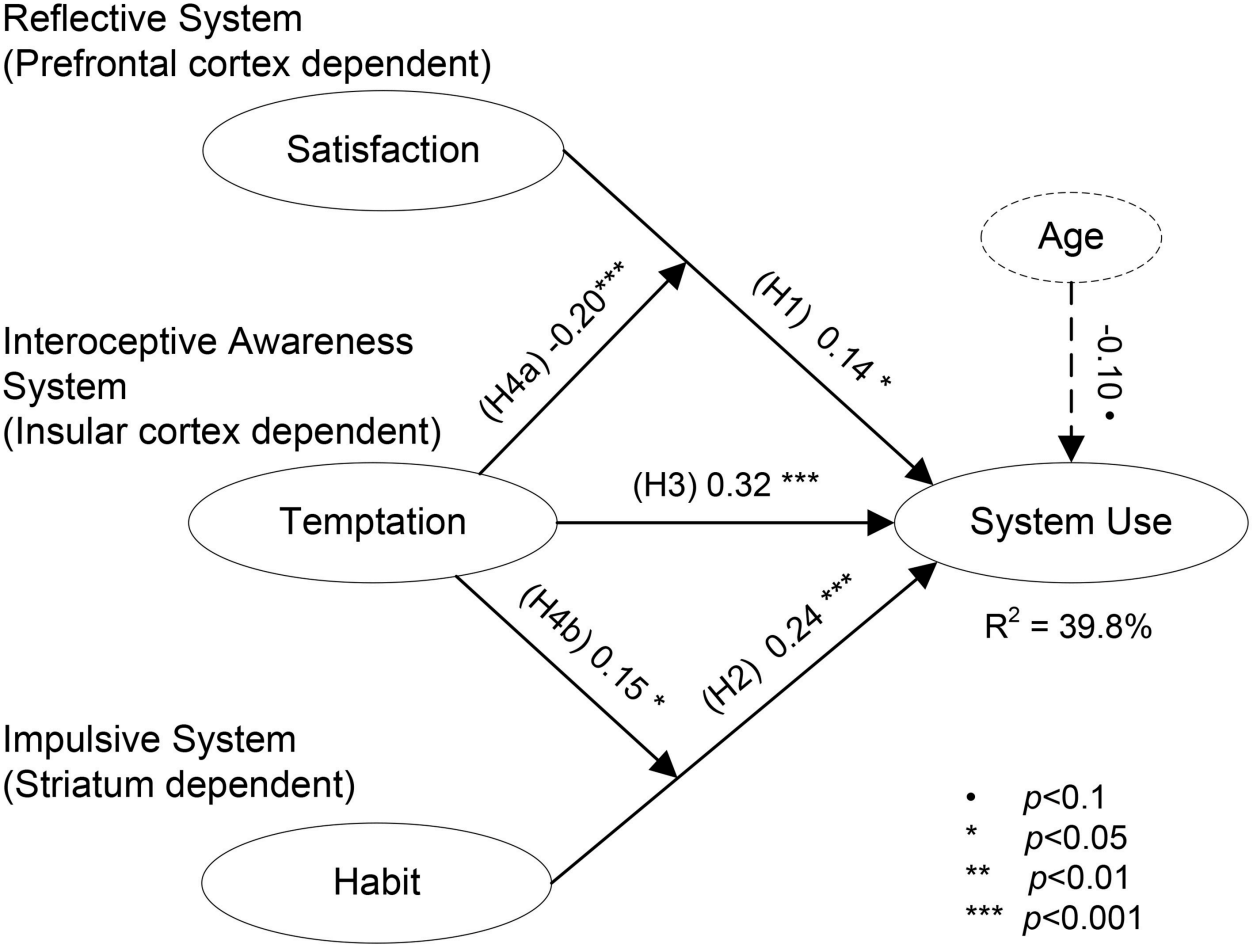
**Structural model—study 1**.

The structural model was estimated in three hierarchical steps. All models included the five control variables (age, gender, contacts on SNS, experience with SNS and GPA). The first (“base”) model included only satisfaction and habit as the known key predictors of system use. These predictors, respectively, represented key activations in the reflective and impulsive brain systems. The second model (“temptation—main effect”) added temptation as another predictor of system use to the first model. It accounts for the effect of temptations, albeit this effect is most likely mediated through reflective and impulsive brain system activations. The third model (“temptation as moderator”) added the hypothesized interaction effects, which were operationalized as the product of the mean-centered averages of the respective items. Table 2 provides the fit indices for the models, the standardized path coefficients and their levels of significance, the variance explained in system use (Squared Multiple Correlation, SMC, similar to *R*^2^ in regression models), and the effect sizes for the added relationships. Figure 2 depicts the temptation-as-moderator model results, including standardized path coefficients, their levels of significance and variance explained values. Additional in-depth moderation analyses are described in Appendix C of Supplementary Materials.

**Table 2:** **Model fit indices, coefficients, explained variances, and effect sizes—study 1^†^, ^††^**.

	**Base model**	**Temptation—main effect model**	**Temptation-as-moderator model**
Fit indices	$\chi_{\left(76\right)}^{2}$ = 115.3, CFI = 0.97, IFI = 0.97, RMSEA = 0.042 with p-close = 0.81, and SRMR = 0.055	$\chi_{\left(135\right)}^{2}$ = 218.2, CFI = 0.95, IFI = 0.95, RMSEA = 0.045 with p-close = 0.75, and SRMR = 0.059	$\chi_{\left(165\right)}^{2}$ = 255.7, CFI = 0.95, IFI = 0.95, RMSEA = 0.043 with p-close = 0.88, and SRMR = 0.057
Satisfaction → use	0.19^**^	0.11 •	0.14^*^
Habit → use	0.36^***^	0.24^**^	0.24^***^
Temptation → use		0.36^***^	0.32^***^
Temptation ^*^ habit → use			0.15^*^
Temptation ^*^ satisfaction → use			−0.20^***^
Age → use	−0.08 ns	−0.09 ns	−0.10 •
Gender → use	0.00 ns	0.35 ns	0.02 ns
Contacts → use	0.10 •	0.07 ns	0.07 ns
Experience → use	0.03 ns	0.04 ns	0.04 ns
gpa → use	−0.06 ns	−0.08 ns	−0.07 ns
*R*^2^ use	25.6%	35.8%	39.8%
*f*^2^ of added constructs–against base model	Baseline	16%	24%

† •p < 0.1, *p < 0.05, **p < 0.01, ***p < 0.001.

†† Effect sizes, f^2^, were calculated using the formula [R^2^ (current model) – R^2^ (baseline)]/[1 – R^2^ (current model)].

The base model in Table 2 indicates that system use can be driven by both reflective and impulsive brain processes; and specifically by presumed activity in the prefrontal cortex (satisfaction assessment) and in the striatum (habit). These two factors explained 25.6% of the variance in system use. The temptation main effect model was used for transition toward the hypothesized moderation model, and for testing H3. While it lends support to H3, the observed direct effect of temptation is likely mediated through reflective and impulsive process, for which this model does not account. We know that the temptation-mediating brain region (the insular cortex) is not directly connected to motor-control regions, and consequently, the observed direct effect should be interpreted as possibly mediated through reflective and impulsive information processing brain regions. This view is accounted for by the third model. This model, “temptation-as-a-moderator,” is consistent with current neuroscience views of this effect (Naqvi and Bechara, 2010)—i.e., that temptations weaken the reflective system effects and strengthen the impulsive system influences. Through this moderation effect, temptations increased system use and led to the execution of less-planned behaviors. The inclusion of the moderation effect increased the variance explained in system use to almost 40%, which is a 55% increase in the variance explained over the base model (25.6%). The moderation effect has a moderate effect size, which indicates that it is quite influential in predicting system use. Lastly, age seems to be marginally influential in the current sample. Regardless of reflective, impulsive, and interoceptive awareness processes, younger users are more likely to use their SNS than older ones.

## Study 2

### Methods

#### Participants

Three hundred sixty nine SNS users who were university students provided complete responses (net response rate of 79%; 90% from those who completed the first survey). The sample included 174 (47%) man and 195 women (53%). Users' average age was 23.2 years (18–47, median = 23, *SD* = 3.83) and they used on average 3.5 SNSs (1–8, median = 3, *SD* = 1.73). Sampled users had an average of 345 contacts on their selected SNS (2–4700, median = 250, *SD* = 474.9) and, on average they had 4.2 years of experience with it (0.25–11, median = 4, *SD* = 2.28). The majority of the sample was Facebook users (57%), followed by Instagram users (31%), Twitter users (6%), and users of other SNS sites such as Google+, LinkedIn, and MySpace (6%).

#### Procedure and materials

Data for study 2 were collected using the same protocol used for collecting data in study 1, but with different measures. The survey items, sources and reliabilities are given in Table 3. This survey captured the same control variables used in study 1, as well as a short-version of the trait self-control scale (Tangney et al., 2004). It was also pretested and shown to be valid (See Appendix A in Supplementary Materials).

**Table 3:** **Study 2 measures and reliabilities**.

	**Sources**	**Items**	
Survey 1 (*t*_1_)	Behavioral expectations (to use the system impulsively; Venkatesh et al., 2008) α = 0.90	(1 = Not at all, 7 = to a very large extent)	
		-I expect to use this social networking website without much planning in the next week	
		-I will use this social networking website based on momentary impulses in the next week	
		-I am likely to use this social networking website without careful thinking in the next week	
		-I am going to use this social networking website spontaneously in the next week	
	Habit (Polites and Karahanna, 2012) α = 0.78	(1 = Strongly disagree, 7 = strongly agree)	
		Lack of awareness α = 0.91	-I choose to use the social networking website without even being aware of (making) the choice
			-I unconsciously start using the social networking website
			-Using the website is something I do subconsciously
		Lack of controllability α = 0.95	-I find it difficult to overrule my impulse to use the social networking website
			-I find it difficult to overcome my tendency to use the social networking website
			-I find it difficult to control my tendency to use the social networking website
			-I find it difficult to restrain my urge to use the social networking website
		Mental efficiency α = 0.90	-Deciding to use this social networking site does not require of me to devote a lot of mental effort
			-Deciding to use this social networking site does not involve much thinking
			-Deciding to use this social networking site requires little mental energy
	Temptation	Identical to study 1; α(4 dimensions) = 0.78, α(bored/idle) = 0.74, α(negative affect) = 0.87, α(positive affect) = 0.79, α(craving) = 0.74	
	Trait self-control (based on Tangney et al., 2004) α = 0.73	Please indicate how much each of the following statements reflects how you typically are: (1 = not at all; 5 = very much)	
		-I am good at resisting temptation	
		-I have an easy time breaking bad habits	
		-I am able to work effectively toward long-term goals	
		I often act after thinking through the alternatives	
Survey 2 (*t*_2_)	Impulsive system use (based on the frequency measure in Turel, 2015a) α = 0.91	Over the previous week, how often did you use the website…(1 = very rarely, 7 = very often)	
		-Spontaneously?	
		-Impulsively?	
		-At the spur-of-the moment?	
		-Without thinking?	
		-Without planning to?	

### Data analysis and results

Data were preliminarily analyzed using the same techniques and tools described in study 1 (see Appendix B in Supplementary Materials). The results demonstrated that the measurement instrument was valid and reliable. Next, a CFA model was estimated using AMOS 23. This model yielded appropriate fit indices: $\chi_{\left(182\right)}^{2}$ = 371.8, CFI = 0.95, IFI = 0.95, RMSEA = 0.053 with p-close = 0.24, and SRMR = 0.044. All loadings were significant (*p* < 0.001). Thus, reasonable convergent and discriminant validities of the measurement model were demonstrated; and implied that it is reasonable to proceed with assessing the structural model.

**Figure 3:**
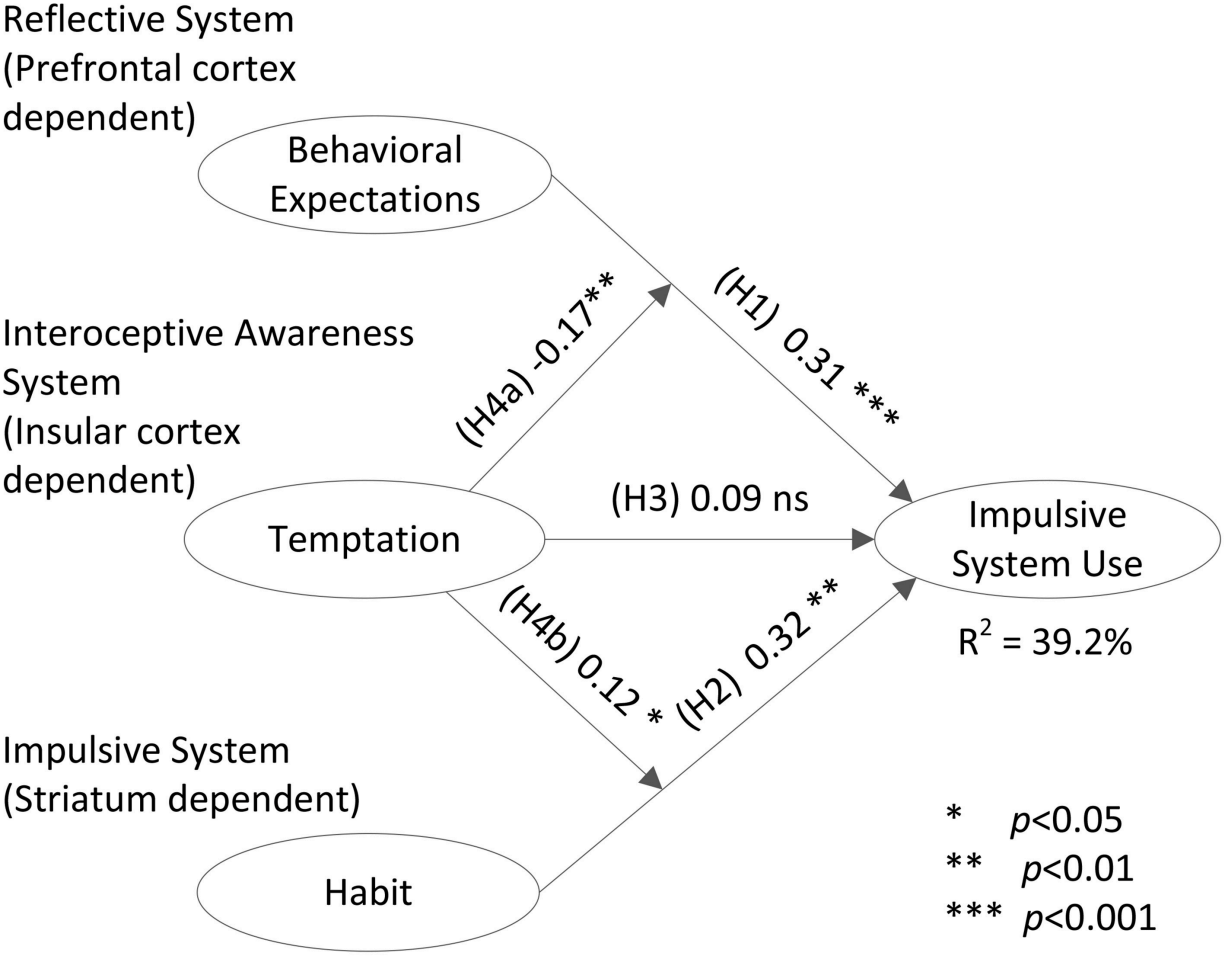
**Structural model—study 2**.

The structural model was estimated using the hierarchical procedure described in study 1. Table 4 outlines the results. Figure 3 depicts the temptation-as-moderator model results for study 2. Additional in-depth moderation analyses are described in Appendix C of Supplementary Materials; they further shed light on how temptations moderate the influences of reflective and impulsive processes on system use behaviors.

**Table 4:** **Model fit indices, coefficients, explained variances, and effect sizes—study 2.^†^, ^††^**.

	**Base model**	**Temptation–main effect model**	**Temptation-as-moderator model**
Fit indices	$\chi_{\left(104\right)}^{2}$ = 239, CFI = 0.95, IFI = 0.95, RMSEA = 0.060 with p-close = 0.1, and SRMR = 0.048	$\chi_{\left(169\right)}^{2}$ = 347.4, CFI = 0.95, IFI = 0.95, RMSEA = 0.054 with p-close = 0.23, and SRMR = 0.049	$\chi_{\left(194\right)}^{2}$ = 375.2, CFI = 0.95, IFI = 0.95, RMSEA = 0.050 with p-close = 0.46, and SRMR = 0.047
Behavioral expectations → impulsive use	0.32^***^	0.32^***^	0.31^***^
Habit → impulsive use	0.26^***^	0.32^**^	0.32^**^
Temptation → impulsive use		0.16 ns	0.09 ns
Temptation ^*^ habit → impulsive use			0.12^*^
Temptation ^*^ behavioral expectations → impulsive use			−0.17^**^
Age → impulsive use	−0.05 ns	−0.05 ns	−0.03
Gender → impulsive use	0.03 ns	0.03 ns	0.03 ns
Contacts → impulsive use	−0.09 ns	−0.07 ns	−0.07 ns
Experience → impulsive use	−0.04 ns	−0.04 ns	−0.04 ns
GPA → impulsive use	0.05 ns	0.03 ns	0.02 ns
Trait self-control → impulsive use	0.06 ns	0.06 ns	0.06 ns
*R*${}_{\text{impulsive use}}^{2}$	35.7%	35.9%	39.2%
*f*^2^ of added constructs—against base model	Baseline	0.3%	5.8%

† *p < 0.05, **p < 0.01, ***p < 0.001.

†† Effect sizes, f^2^, were calculated using the formula described in study 1.

Table 4 demonstrates similar picture to the one portrayed in study 1, and hence strengthens our confidence in the reflective-impulsive-interoceptive awareness idea. The base model shows that impulsive system use can be predicted by expectations, as well as driven by habit. These two factors explained 35.7% of the variance in system use. The results of the temptation main effect model differed from the ones observed in study 1. In the current study, the effects of the reflective and impulsive factors remained significant, but temptation did not exert a direct effect on system use; which is aligned with the neuro-scientific evidence regarding the role of insular activity in decision making (Naqvi et al., 2007; Naqvi and Bechara, 2009, 2010). Hence, H3 was not supported in this study. Lastly, the “temptation-as-a-moderator” model lent support to H4a and b. Control variables exerted no significant effects in the current study. This study explained again, almost 40% of the variance in the outcome variable.

## General discussion

What determines the extent to which people use hedonic information systems (impulsively or not)? Can these perceptions and states be conceptually linked to the same brain processes and system which underlie problematic and addictive behaviors? Is there preliminary evidence in support of a recent tripartite conceptualization of the brain etiology of problematic and addictive behaviors? The findings of two studies indicate that in line with dual-system theories, technology use is driven by perceptions and states that may be reasonably linked to at least two information processing faculties. The first one is reasoned-action as captured by the reflective system, and operationalized in this study as satisfaction and behavioral expectation. The second is the impulsive system which activates cue-action links through mental associations. In both studies 1 and 2 this system focused on habit.

This study extended this reflective-impulsive view and included presumed behavioral effects of the interoceptive awareness brain system. Specifically, it focused on temptations which have the capacity to operate outside of the reasoned-action system and influence behavior (Baumeister, 2002; Hofmann et al., 2008) through suppressing reflective processes, and amplifying impulsive ones (Naqvi et al., 2007; Naqvi and Bechara, 2009). As expected and in line with recent neurocognitive views of problematic and addictive behaviors, temptations had a significant influence on system use. Adding temptation as a moderator to the simplified reflective-impulsive model increased the variance explained in system use from 25.6% to almost 40% in study 1 and the variance explained in impulsive system use from 35.7% to almost 40% in study 2. This implies that technology use behavior, while at its heart is driven by reflective-processes and sometimes by habit, can also often be influenced by salient situational temptations, which violate the balance between users' reflective and impulsive information processing processes.

Several implications of these findings should be noted. First, the two studies illuminate the importance of a third relatively less-explored brain system, namely the interoceptive awareness system. As the results of both studies demonstrate, certain internal desires, such as temptations, can cause decision makers to shift away from homeostasis, and generate disequilibrium between the reflective and impulsive information processing systems. Specifically, when such aware sense of desire is strong, it weakens one's reflective system influences and strengthens his or her impulsive system effects. Hence, our findings provide a behavioral account for the neuroscience theory put forth by Noël et al. (2013).

Second, this study extends the traditional view of IS users as reasoned decision makers and conceptually and empirically demonstrates that IS use can also have less-planned and less-controllable elements. This suggests that existing rationale-based IS use models (Bhattacherjee, 2001), which are mostly based on the reflective system, are valid. Yet, they represent only a part of what is going on in users' minds. As per the reflective-impulsive model, users also have an impulsive information processing system that has been accounted for through the examination of habits. Both studies indicated that presumed impulsive system activations (habits) are a significant predictor of system use and that oftentimes; this system exerts stronger effects on use than the select reflective processes do, at least in the case of SNS. It is therefore possible that SNS use is strongly driven by the impulsive system (habituation), and is less planned than assumed. In study 2, the notion of less-planned, spontaneous and impulsive use was directly examined. A mean score of 4.38 on a 1–7 scale indicates that impulsive use is quite prevalent in SNS settings (people engage in it quite often), and hence it merits further research. It can perhaps explain the rise in addiction-like symptoms observed among many SNS users (Turel et al., 2014; Turel, 2015a) and the increase in problematic behaviors among such users (Turel and Bechara, 2016).

Third, both studies shed light on the specific situations that tempt users to engage in SNS use, and provide a consistent view across the samples, which strengthens our confidence in these findings. The tempting circumstances fall into positive affect, negative affect, craving and idle time or boredom situations. As per the means given in Tables 5, 6 in Appendix B of Supplementary Materials, the most tempting situation, at least in the current samples, is when people are bored or have idle time. Three paired-sample *t*-tests were performed with study 1 data (*t* = 12.3, 15.2, and 8.4; all with *p* < 0.001) revealed that the mean of this type of temptation is higher than those of the other three types of temptations. Similar results are obtained when examining study 2 data (*t* = 14.6, 15.9, and 7.1; all with *p* < 0.001). These results imply that boredom or idle time, as was often indicated by the focus group we used for scale development, is potentially a key driver of SNS use, and that it does so through creating a strong sense of temptation, which weakens rational reflections, and promotes rash behaviors. Nevertheless, this result may not be generalizable to other IS, not just in terms of magnitude, but also in terms of the specific tempting situations that may drive system use. Different subjects of temptation may result in different ranges of tempting situations (Marlatt, 1996; El-Sheikh and Bashir, 2004; Marlatt and Donovan, 2005). Thus, future studies may explore the temptations developed in other potentially-tempting IS use contexts and incorporate them into the interoceptive awareness system in IS use models.

Fourth, study 1 showed that SNS use can be driven in part by impulsive processes; and study 2 showed that the rash, less-planned system use is quite common and can be studied as a standalone phenomenon. Given the prevalence of impulsive and temptation-driven system use which was demonstrated in both studies, it is interesting to note that such system use can, in some cases, hurt the viability of long-term important goals, such as succeeding in school (e.g., when tempted to play videogames instead of studying) or staying alive (e.g., when tempted to respond to an SNS post while driving). Thus, future research may examine ways to reduce temptations or increase users' abilities to resist them in such situations.

Fifth, Table 6 (Appendix B in Supplementary Materials) shows that younger users can feel stronger temptations than older ones. This is similar to the age differences observed in other contexts focusing on rewarding, problematic or risky behaviors, and addictions; and may stem from stages in one's biological development (Somerville and Casey, 2010). Another possible explanation is that the amount of idle time may decrease with age, precipitously declining beyond the emerging adult years, and hence older users may face less tempting situations than younger users. Similarly, Tables 5, 6 (Appendix B in Supplementary Materials) also consistently show that men, at least in our samples, were less tempted to use SNS than women. Perhaps it is a feature of the samples. Alternatively, it may stem from sex-based differences in neural activity (Lighthall et al., 2012), hormonal levels related to decision making (Allen et al., 1991; Gur et al., 1995, 1999), and insular cortex volume and activity (Garavan, 2010; Tanabe et al., 2013). The roles of age and sex in the reflective-impulsive-interceptive awareness model should therefore be further explored in future research.

Lastly, this study's findings have interesting potential practical implications. While in some cases unplanned use of SNS may be healthy and beneficial (e.g., it may allow a person to “recharge” his or her mental resources and/or cope with boredom), in many other cases it can be unhealthy and harmful for a person's long term goals (e.g., when it is done in improper situations such as while in class or driving). In such situations, it may be desirable to reduce, control or prevent system use. Preventing unplanned, temptation-driven system use in such cases is in part the responsibility of users. They should be aware of the role of temptations in their decision making, and learn how to appropriately deal with them and potentially resist them, when they identify the use situation to be problematic. To this end, users can train themselves to activate the long-term goals they may infringe by yielding to temptations, and this should help them fight the temptation (Fishbach et al., 2003; Fishbach and Shah, 2006). For instance, whenever tempted to use an SNS in class, students may remind themselves that this is bad for their academic achievement. They may also remove situational cues that activate the temptations. For example, a student that has dorm mates who play videogames may transfer himself or herself to a different dorm to avoid these temptation-driving cues. In extreme cases where users sense very strong temptations in inappropriate use situation and they may feel they cannot control them, possibly when addiction conditions develop (Ryan et al., 2014), several treatment options are possible such as psychosocial therapy (Anton et al., 1999) and deep brain stimulation (Hanlon et al., 2012), though further research on the merit of such techniques is needed.

Two key limitations of this study which point to future research should be acknowledged. First, the operationalization of the impulsive, reflective and interoceptive-awareness systems in the two studies was rather simplistic. This was done because the objective was to introduce and illuminate the importance of these systems and their interactions in general and impulsive IS use processes. Accordingly, each study we describe here focused on a single, yet arguably central construct presumed to be dependent on each one of these systems. Each system, though, can be more complex and cover many other activations and associated perceptions and states; and it is practically impossible to account for all of them in a single study. The use of two studies with different presumed activations and measures of these systems strengthens the confidence in the tripartite reflective-impulsive-interceptive awareness conceptualization. Nevertheless, future research may use the studies we describe in this manuscript as platforms for expanding the presumed activations which are dependent on the reflective, impulsive and interoceptive awareness systems, and account for more complex interactions and influences.

Second, a clear mapping of constructs onto brain systems was assumed because these brain systems were found to be necessary for the described processes (necessity often determined in lesion studies—when the discussed brain region is wiped-out, the process is impaired). For example, the insula is necessary for interceptive awareness (Naqvi et al., 2007), but many other brain regions may be involved in this process; e.g., the visual cortex needs to translate visual cues into temptations, and memory and self-concept functions may be activated too. Hence, it should be noted that no such clear mapping from constructs to brain regions exists, because reflective, impulsive, and interoceptive awareness processes activate many brain systems which are interconnected. Similarly, the brain systems we emphasized in this study as involved in reflective-impulsive-interoceptive awareness process are involved in many other tasks and assessments. For instance, the amygdala is involved in generating prime emotions (Bechara et al., 2000a) and not just in the examined impulsive activations. Consequently, readers should not interpret the proposed behavioral-neural associations as one-to-one relationships, and many fMRI studies may be needed to shed further light on the many regions and connectivity channels which may be involved in decision processes in the case of information systems. Nevertheless, this study provides first strides into understanding the potential etiology of problematic IS use behaviors. Hence future fMRI-based studies can augment, supplement and refine the findings described in this manuscript.

## Author contributions

OT participated in study conceptualization, analysis and write-up. AB participated in study conceptualization and write-up.

### Conflict of interest statement

The authors declare that the research was conducted in the absence of any commercial or financial relationships that could be construed as a potential conflict of interest.
