# Supplementary material for: A Triadic Reflective-Impulsive-Interoceptive Awareness Model of General and Impulsive Information System Use: Behavioral Tests of Neuro-Cognitive Theory
Source: Front Psychol. 2016 Apr 26;7:601. doi: 10.3389/fpsyg.2016.00601 (PMC4845517; doi:10.3389/fpsyg.2016.00601)
Supplement: Supplementary file 1 [file Table1.doc]

Modern day addictions: The role of temptations in a reflective-impulsive-interoceptive awareness model of information system use

APPENDIX A –Preliminary Validation of Measurement Instruments

## Study 1

## The temptation measure we used was developed in the context of smoking, and originally included three dimensions of one’s level of temptation to smoke cigarettes: in negative affect situations, in social and positive affect situations, and in craving situations. It was assumed, consistent with the conceptualization proposed by Velicer et al. , that a person has a general level of temptation to engage in the presumed-to-be rewarding studied behavior (e.g., smoke a cigarette, use SNS) across situations. This general temptation is manifested through the level of temptation a person feels in a range of prototypical use situations that provide him or her with strong interoceptive signals (i.e., cues) to engage in the target behavior (i.e., behavior schemata).

While this measure is well established and has been validated, we felt that given differences between its original application area (smoking) and the current application area (using SNS), its adaptation should be re-examined and re-validated. We first asked a focus group of 15 SNS users (students) to describe situations in which they may be tempted to use their SNS. Their responses fell into the three existing categories of tempting situations (e.g., negative affect = when I break up with my boyfriend; positive affect = when I pass a difficult exam; craving = when I wake up), but also pointed to the fact that when they are bored (e.g., in class, home alone, or when stuck in traffic) they feel strong temptations to use their SNS. That is, they identified boredom during idle time as a key situation in which SNS use temptations are generated. The literature also points to the fact that boredom during idle time is a possible tempting situation in which SNS use may be stimulated . Thus, a measure of boredom/idle time based on Gwaltney et al. and the situations the focus groups have identified was adapted to the SNS context, and added as a fourth manifestation of one’s temptation. Such context-specific extensions of temptation measures are quite common, because each tempting target behavior is slightly different and can be manifested in different situations and given different contextual cues. For example, for drug users, physical discomfort is a situation which may produce temptation to use drugs; and this situational dimension was not included in the basic three-dimensional conceptualization of temptation .

In order to test the viability of the expanded temptation measure, it was presented to seven experts- three university professors, two psychologists, one social media professional, and two cognitive neuroscientists. They were asked to comment on the face and content validity of the measure. All respondents reaffirmed its face and content validity. The scale was also presented to 12 university students (not from the classes from which other samples were taken). For each item (situation) they were asked to report on a scale of 1 (none) to 4 (many) how many SNS users they know who will be tempted to use their SNS in this situation. The average for items ranged from 2.63 to 3.91, indication that the described situations will entice at least some individuals if not many to use their SNS. Especially the Boredom/Idle Time dimension seemed to be a good addition to the existing three dimensions because it seems to capture a set of very tempting scenarios (average of all boredom/idle time items = 3.36).

Next, the scale’s quantitative reliability and validity properties were assessed. To this end, another 30 respondents (not from the classes from which other samples were taken) were asked to rate their temptation to use their SNS using the provided scale. All four dimensions were reliable with Cronbach’s alphas ranging from 0.79 to 0.93. Furthermore, principal component analysis produced four components with the expected loading pattern, which explained 80% of the variation in the data. Overall, both the qualitative and quantitative pre-test results lend preliminary support to the SNS temptation scale.

## Study 2

In study 2 we used validated behavioral expectation and system use measures which were adapted to the impulsive system use context. We again felt that it is worthwhile to validate such adaptations. To do so, the measures were first presented to five IS scholars without disclosing the scales’ content domain and in a randomized order of items. All scholars were able to group the items for each construct together and did identify that the first captures expectations regarding impulsive unplanned use, and that the second captures impulsive unplanned use. Afterwards, the items were pretested with a sample of 30 students at two points in time. Both scaled loaded on separate factors (loadings of 0.90-0.93 of behavioral expectation items, and loadings of 0.78-0.86 of the impulsive use items), and were uni-dimensional and reliable (α=0.94 for behavioral expectations, and α=0.92 for impulsive use). The factor scores of behavioral expectations at t_1_ predicted impulsive use at t_2_ (β=0.60, p<0.000). These preliminary tests provide confidence regarding the content and predictive validities, as well as regarding the dimensionality and reliability of these concepts.

# APENDIX B – DETAILED Validity and Reliability Assessments- Main Studies

## Study 1

First, because data pertained to a cross-section of SNSs, the possibility of SNS-based differences was examined. To this end, a multivariate analysis of variance (MANOVA) model was estimated using SPSS 23: The research model’s constructs were included as dependent variables, the selected SNS was a fixed factor, and age, gender, contacts, years of SNS experience, and GPA, were used as covariates. The model has yielded non-significant Pillai’s Trace indices (for the selected SNS it was 0.08, F=1.4, p < 0.13). This demonstrated that there were no omnibuses SNS-type-based differences in the data. Hence, the dataset was analyzed as a whole.

Next, several aspects of measure validity were examined. Table 5 outlines descriptive statistics and reliability measures for all latent variables and controls, as well as inter-construct correlations. As can be seen, all of the measures were reliable with Cronbach’s alphas, composite reliability and square root of Average Variance Extracted (AVE) scores over 0.7. This suggested sufficient convergent validity. In addition, the square root of the AVE for each first order construct exceeded the correlations of the construct with other constructs. This demonstrated sufficient discriminant validity . Overall, it was concluded that the measures are reliable and present sufficient discriminant and convergent validities.

Table 1: Latent Variable Descriptive Statistics, Correlations and Reliability Indices – Study 1^†,††^

|  | Mean (SD) | (1) | (2) | (3) | (4) | | (5) | (6) | (7) | | (8) | (9) | (10) | (11) | | (12) | (13) |
| --- | --- | --- | --- | --- | --- | --- | --- | --- | --- | --- | --- | --- | --- | --- | --- | --- | --- |
| (1) Satisfaction | 5.14 (0.89) | .86 (.86) [.78] | | | |  | | | | | | | | | | |  |
| (2) Habit | 5.20 (1.05) | .38** | .78 (.81) [.77] | | | | | | |  | | | | | | | |
| (3) Temptation (2^nd^ Order) | 2.81 (0.59) | .30** | .35** | .72 (.73) [.72] ^†††^ | | | | | | |  | | | | | | |
| (4) Temptation when Bored/ Idle | 3.29 (0.80) | .26** | .30** | .70** | .71 (.73) [.70] | | | | | | |  | | | | | |
| (5) Temptation in Negative Affect Situations | 2.40 (0.91) | .15** | .16** | .77** | .29** | | .83 (.84) [.76] | | | | | |  | | | | |
| (6) Temptation in Positive Affect Situations | 2.64 (0.75) | .15** | .25** | .68** | .29** | | .39** | .79 (.81) [.71] | | | | | |  | | | |
| (7) Temptation in Craving Situations | 2.93 (0.72) | .33** | .34** | .79** | .50** | | .51** | .37** | .71 (.81) [.71] | | | | | | |  | |
| (8) System Use | 3.55 (0.85) | .33** | .36** | .40** | .33** | | .27** | .20** | .39** | | .76 (.74) [.70] | | |  | | | |
| (9) Age | 22.77 (3.72) | -.07 | -.11* | -.07 | -.06 | | -.09 | .02 | -.06 | | -.13* | NA | | |  | | |
| (10) Gender (Male=1) | NA | -.10 | -.10 | -.15** | -.11 | | -.05 | -.16** | -.13* | | -.05 | .15** | NA |  | | | |
| (11) Contacts on SNS | 428.70  (478.07) | .11* | .09 | .13* | .14* | | .07 | .01 | .15** | | .15** | -.13* | .03 | NA | | |  |
| (12) Years of Experience on SNS | 4.54 (2.77) | -.09 | .02 | -.01 | -.06 | | .03 | .02 | -.04 | | .02 | .00 | .05 | -.02 | | NA | |
| (13) GPA | 3.73 (1.48) | .04 | -.02 | .04 | -.04 | | .06 | -.00 | .10 | | -.02 | -.07 | -.15** | -.04 | | -.03 | NA |

† On the diagonal: Cronbach’s Alpha, (Composite Reliability) and [square root of AVE]

††* *p* < 0.05 ** *p* < 0.01

††† For the second-order construct the reliability indices were calculated using the averages of the first order constructs as reflective indictors.

## Study 2

First, possible SNS-based differences were examined with MANOVA model similar to the one used in study one. The results indicated no significant omnibus differences based on SNS (Pillai’s Trace of 0.78, F=1.7, p < 0.11). Hence, data were analyzed as a whole. Next, a correlation matrix was generated, and descriptive statistics, Cronbach’s alphas, composite reliability and square root of AVE scores were calculated for the model’s constructs. These are presented in Table 6. As can be seen, all Cronbach’s alphas, composite reliability and square root of Average Variance scores surpassed the 0.7 threshold. This implies sufficient convergent validity. Moreover, sufficient discriminant validity was demonstrated by the fact that the square root of the AVE for each first order construct exceeded the correlations of the construct with other constructs. Overall, the measures appeared to be valid and reliable.

Table 2: Latent Variable Descriptive Statistics, Correlations and Reliability Indices- Study 2^†,††, †††^

|  | Mean (SD) | (1) | (2) | (3) | (4) | (5) | (6) | (7) | (8) | (9) | (10) | (11) | (12) | (13) | (14) | (15) | (16) |
| --- | --- | --- | --- | --- | --- | --- | --- | --- | --- | --- | --- | --- | --- | --- | --- | --- | --- |
| (1) Behavioral Expectations | 5.07  (1.39) | .90 (.90) [.84] | | | | | | | | | | | | | | | |
| (2) Habit (2^nd^ Order) | 4.32  (1.10) | .42** | .72 (.75) [.70] | | | | | | | | | | | | | | |
| (3) Habit- Awareness | 3.92  (1.73) | .30** | .80** | .91 (.91) [.88] | | | | | | | | | | | | | |
| (4) Habit- Controllability | 3.58  (1.60) | .29** | .78** | .45** | .95 (.96) [.92] | | | | | | | | | | | | |
| (5) Habit- Efficiency | 5.45  (1.24) | .32** | .59** | .12* | .13* | .90 (.90) [.86] | | | | | | | | | | | |
| (6) Temptation (2^nd^ Order) | 2.83  (0.67) | .44** | .51** | .42** | .48** | .14** | .78 (.79) [.73] | | | | | | | | | | |
| (7) Temptation when Bored/ Idle | 3.27  (0.85) | .45** | .43** | .32** | .36** | .21** | .76** | .74 (.79) [.75] | | | | | | | | | |
| (8) Temptation in Negative Affect Situations | 2.42  (1.01) | .24** | .31** | .25** | .32** | .05 | .76** | .40** | .87 (.88) [.81] | | | | | | | | |
| (9) Temptation in Positive Affect Situations | 2.63  (0.77) | .26** | .39** | .39** | .38** | -.01 | .74** | .46** | .38** | .79 (.80) [.72] | | | | | | | |
| (10) Temptation in Craving Situations | 3.00  (0.74) | .44** | .51** | .39** | .46** | .18** | .77** | .61** | .50** | .53** | .74 (.80) [.71] | | | | | | |
| (11) Impulsive System Use | 4.38  (1.43) | .49** | .42** | .32** | .32** | .24** | .39** | .36** | .21** | .27** | .40** | .91 (.92) [.84] | | | | | |
| (12) Age | 23.2  (3.83) | -.11* | -.15** | -.13** | -.10* | -.08 | -.12* | -.14** | -.09 | -.03 | -.12* | -.15** | NA |  | | | |
| (13) Gender (Male=1) | NA | -.06 | -.10* | -.11* | -.09 | -.00 | -.17** | -.15** | -.13** | -.13* | -.13* | -.10* | .07 | NA |  | | |
| (14) Contacts on SNS | 345.2  (474.8) | .18** | .21** | .13** | .23** | .08 | .22** | .18** | .14** | .12* | .26** | .08 | -.16** | .01 | NA |  | |
| (15) Trait Self-Control | 4.20  (2.28) | -.02 | -.08 | -.06 | -.19** | .12* | -.05 | -.02 | -.06 | -.08 | .02 | -.04 | -.07 | .06 | .09 | .73 (.80) [.71] | |
| (16) Years of Experience on SNS | 3.68  (1.54) | .07 | .02 | -.02 | .09 | -.03 | .04 | .01 | .06 | -.01 | .05 | -.01 | .10* | .00 | .05 | -.09 | NA |
| (17) GPA | 3.44  (0.62) | -.02 | -.11* | -.11* | -.08 | -.02 | -.07 | -.15** | .02 | -.11* | -.01 | .01 | -.05 | -.02 | -.06 | .10* | .01 |

† On the diagonal: Cronbach’s Alpha, (Composite Reliability) and [square root of AVE]

††* *p* < 0.05 ** *p* < 0.01

††† For the second-order construct the reliability indices were calculated using the averages of the first order constructs as reflective indictors.

# APENDIX C – Moderation Analyses

## Study 1

The significance of the interaction terms in the structural model demonstrated that, as hypothesized, temptations weaken the effect of satisfaction assessments, which represent prefrontal cortex, reflective, activity; and strengthen the effect of habit, which represents dorsal-striatum, impulsive activity. In order to further explore these moderation effects, they were plotted using the Interaction software package[[1]](#footnote-2) with mean-centered predictors. The resultant plots are presented in Figure 4. The implied path coefficient and its two-tailed level of significance are reported next to each regression line.

The left-panel plot shows the positive relationship between satisfaction and system use, and how it is weakened as temptations grow. This process takes place up to the point where satisfaction becomes non-significant (approximately when temptations reach two standard deviations above the mean), presumably due to the fact that the insular-cortex activity “hijacks” the prefrontal cortex and mutes reflective processes, such as assessing satisfaction and using this information for decision making.

|  |  |
| --- | --- |

Figure 1: Interaction Plots – Study 1

The right-panel plot shows the positive relationship between habit and system use, and how it is amplified by growth in the strength of one’s temptations. At low levels of temptations (e.g., when one’s habit is not appealing or intrinsically rewarding; two standard deviations below the temptation mean), habit does not influence system use. The observed growth in habit effect as a function of temptations is presumably due to the fact that the insular-cortex activity “excites” the striatal system and exacerbates impulsive processes, such as habitual behaviors. Both moderating effects support the role of interoceptive awareness we portray in this study, as well as the tripartite model we introduce.

## Study 2

In order to gain a better insight into the moderating effects of temptations, moderation analysis was performed using the procedures and tools described in the study 1 part of this appendix. The results are depicted in Figure 5. The left-panel plot shows the positive relationship between behavioral expectations and impulsive system use, and how it is weakened as temptations grow. When behavioral expectations reach two standard deviations above the mean they start becoming a non-significant predictor of impulsive system use. The right-panel plot shows the positive relationship between habit and impulsive system use, and how it grows when temptations increase. Both moderating effects support the role of interoceptive awareness we portray in this study, as well as the reflective-impulsive-interceptive awareness model we introduce.

|  |  |
| --- | --- |

Figure 2: Interaction Plots – Study 2

# References- Appendices

El-Sheikh, S.E.-G., and Bashir, T.Z. (2004). High-risk relapse situations and self-efficacy: comparison between alcoholics and heroin addicts. Addictive Behaviors 29, 753-758.

Fornell, C., and Larcker, D. (1981). Evaluating structural equation models with unobservable variables and measurement error. Journal of Marketing Research 18, 39-50.

Gwaltney, C.J., Shiffman, S., Norman, G.J., Paty, J.A., Kassel, J.D., Gnys, M., Hickcox, M., Waters, A., and Balabanis, M. (2001). Does smoking abstinence self-efficacy vary across situations? Identifying context-specificity within the relapse situation efficacy questionnaire. Journal of Consulting and Clinical Psychology 69, 516-527. doi: 10.1037//0022-006x.69.3.516.

Hew, K.F. (2011). Students' and teachers' use of Facebook. Computers in Human Behavior 27, 662-676. doi: 10.1016/j.chb.2010.11.020.

Sheldon, P. (2008). The relationship between unwillingness-to-communicate and students' Facebook use. Journal of Media Psychology: Theories, Methods, and Applications 20, 67-75.

Velicer, W.F., Diclemente, C.C., Rossi, J.S., and Prochaska, J.O. (1990). Relapse situations and self-efficacy: An integrative model. Addictive Behaviors 15, 271-283. doi: 10.1016/0306-4603(90)90070-e.

1. See www.danielsoper.com [↑](#footnote-ref-2)
